# Cell Division Cycle 42 plays a Cell type-Specific role in Lung Tumorigenesis

**DOI:** 10.1038/s41598-017-10891-0

**Published:** 2017-09-04

**Authors:** Chao Zheng, Yuetong Wang, Liu Yang, Shuhua Zhou, Yijun Gao, Fuming Li, Yan Feng, Zuoyun Wang, Lixing Zhan, Qin Yan, Xueliang Zhu, Kwok-Kin Wong, Zhengjun Chen, Hongbin Ji

**Affiliations:** 1 State Key laboratory of Cell Biology, Shanghai Institutes for Biological Sciences, Chinese Academy of Science, Shanghai, 200031 China; 2 CAS center for Excellence in Molecular Cell Science, Shanghai Institutes for Biological Sciences, Chinese Academy of Science, Shanghai, 200031 China; 3 Innovation Center for Cell Signaling Network, Shanghai Institutes for Biological Sciences, Chinese Academy of Science, Shanghai, 200031 China; 4 National Center for Protein Science Shanghai, State Key laboratory of Molecular Biology, Institute of Biochemistry and Cell Biology, Shanghai Institutes for Biological Sciences, Chinese Academy of Science, Shanghai, 200031 China; 5 Shanghai Science Research Center, Chinese Academy of Sciences, Shanghai, 201204 China; 6 Institute for Nutritional Sciences, Shanghai Institute for Biological Sciences, Chinese Academy of Sciences, Shanghai, 200031 China; 7 Department of Pathology, Yale University School of Medicine, New Haven, CT 06510 USA; 8 Department of Medical Oncology, Dana-Farber Cancer Institute, Boston, Massachusetts, 02115 USA; 9 Belfer Institute for Applied Cancer Science, Dana-Farber Cancer Institute, Boston, Massachusetts, 02115 USA; 10 School of Life Science and Technology, Shanghai Tech University, Shanghai, 200120 China

## Abstract

Cell division cycle 42 (CDC42) plays important roles in polarity establishment and maintenance as well as cell cycle progression and cell division. Although disruption of cell polarity is a prerequisite in epithelial tumor initiation, the roles of CDC42 in tumorigenesis are still poorly understood. Here we find that *Cdc42* deficiency inhibits the *Kras* ^*G12D*^-induced lung alveoli tumor formation, while conversely promotes bronchiole tumor formation in mice. Bronchial *Cdc42* loss destroys contact inhibition potentially through cell polarity disruption, and results in increased tumor formation. In contrast, deletion of *Cdc42* in alveoli cells prevents *Kras* ^*G12D*^-induced cell proliferation, which leads to reduced tumor formation. Further analyses of clinical specimens uncover a significant positive correlation between CDC42 and type II alveolar epithelial cells marker SP-A, indicating the potential importance of CDC42 in this specific subset of lung cancer. Collectively, we identify the lineage-specific function of CDC42 in lung tumorigenesis potentially through the regulation of cell polarity integrity.

## Introduction

The Rho GTPase family member CDC42 plays an essential role in regulating multiple cellular processes including cell proliferation, division, migration, morphogenesis, and especially epithelial polarity establishment. In its GTP-bound form, CDC42 initiates actin polymerization, filopodia formation by binding to PAK family of serine/threonine kinases and WASP, and formation of epithelial junctions by binding to PAR6, causing followed phosphorylation of aPKC targets^1^.

Deregulation of CDC42 may result in multiple cellular defects and significantly contribute to cancer formation^1, 2^. CDC42 was initially thought to be oncogenic in several cancer types via contributing to cell proliferation, survival, migration and invasion^2–4^. While gene mutations have not been detected in human cancers^5^, CDC42 has been reported to be overexpressed in several types of human cancers including colorectal adenocarcinoma^6^, breast cancer^7^, testicular cancer^8^ and non-small cell lung cancer^9^. Elevated CDC42 level may also be detrimental to patient survival as overexpression of CDC42 in melanoma positively correlated with prognostic indicators^10^. Constitutive activated CDC42 is capable of inducing immortalized fibroblasts transformation^11^ and functional CDC42 is required for *HRas* ^*V12*^ induced colony formation of NIH-3T3 cells in soft agar^12^.

Interestingly, recent studies based on *Cdc42* conditional knock-out mice have uncovered a tumor suppressive role of *Cdc42*, showing that hepatocyte-specific deletion of *Cdc42* results in chronic liver damage, hepatomegaly and development of hepatacellular carcinoma^13^. Also, induced gene targeting of *Cdc42* in murine bone marrow hematopoietic stem/progenitor cells results in a loss of hematopoietic stem cell quiescence and hyperproliferation of blood progenitors^14^. Consistently, neuroblastomas with N-myc amplification display deletions of the short arm of chromosome 1 containing the *Cdc42* gene in 90–95% of cases, and one copy of *Cdc42* is consistently lost in this type of cancer^15^. These data suggest that the role of *Cdc42* as oncogene or tumor suppressor might be lineage dependent^16^.

Lung cancer is one of the most devastating diseases worldwide with different subtypes derived from trachea, bronchiole or peripheral alveoli. Previous studies have detected high CDC42 expression in human lung cancer samples^9^ and cell lines^17^ and demonstrate its contribution to cancer cell migration. Moreover, down-regulation of CDC42 is found to inhibit lung cancer cell growth^18^ and invasiveness^17, 19–22^. CDC42 also promotes trans-endothelial migration of lung cancer cells through β1 integrin^23^. These observation are consistent with oncogenic role of CDC42.

Here through detailed studies of *Cdc42* deletion in distinct cell types using lineage specific promoter driven CRE in *Kras* ^*G12D*^ driven lung cancer mouse model, we have identified both tumor-promoting and tumor-suppressive function of CDC42 in type II alveolar epithelial cells and Club cells, respectively. Our data further show that CDC42 prevents lung bronchiole tumor formation potentially through regulation of cell polarity integrity. In accordance with its tumor promoting role in alveolar tumor formation, CDC42 expression is positively correlated with alveolar marker surfactant protein A1 (SP-A) expression in human lung adenocarcinoma patients.

## Results

### *Cdc42* loss promotes bronchiole tumor formation but inhibits alveoli tumor formation in *Kras* mouse model

**Figure 1:**
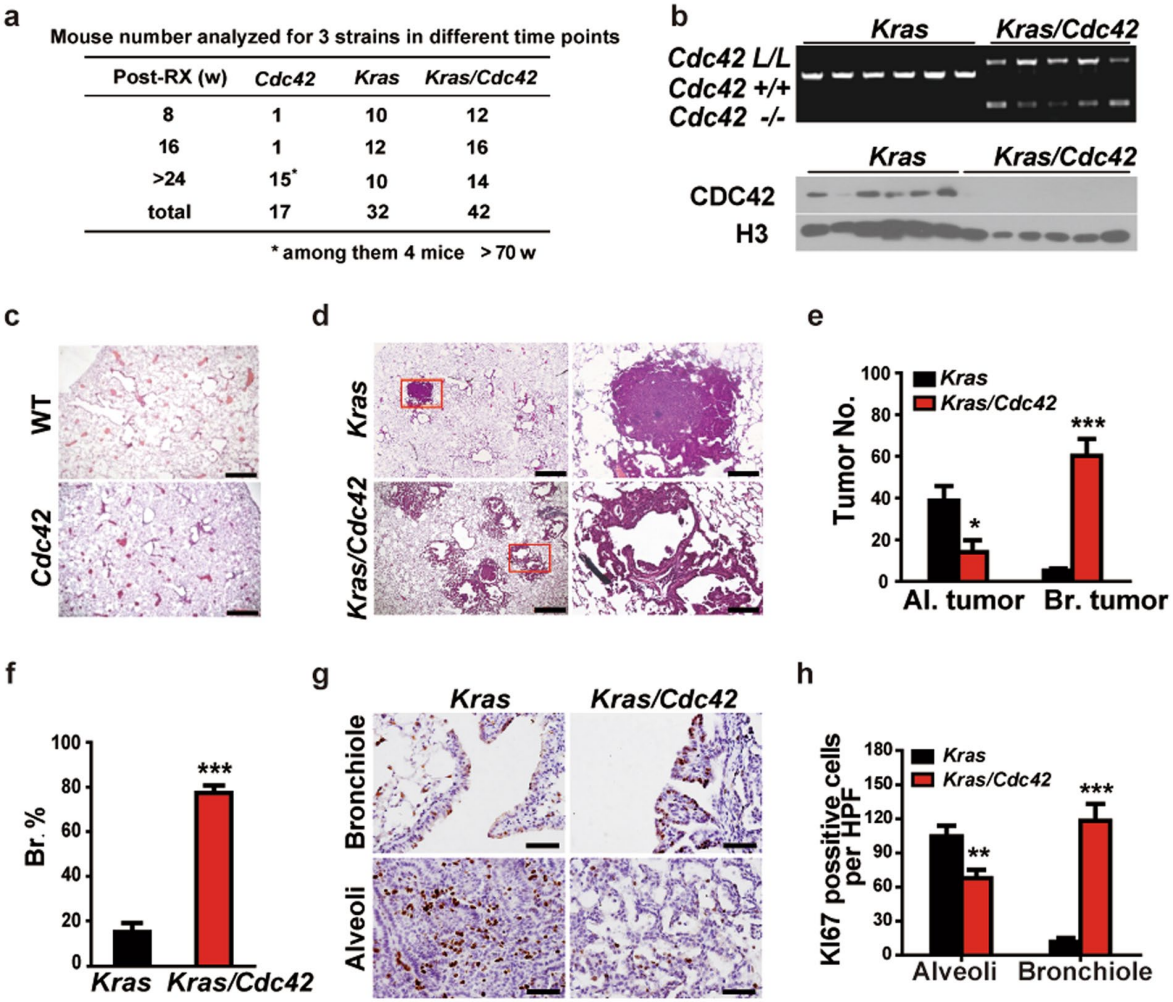
*Cdc42* loss promotes bronchiole tumor formation but inhibits alveoli tumor formation in *Kras* mouse model. (**a**) Mouse number analyzed for 3 strains in indicated time points. (**b**) Up: PCR analysis of conditional *Cdc42* allele recombination in tumors from *Kras* and *Kras/Cdc42* mice; Bottom: Western blot of CDC42 expression in tumors from *Kras* and *Kras/Cdc42* mice. Histone 3 (H3) serves as a loading control. The cropped blots are used in the figure. The membranes were cut prior to exposure so that only the portion of gel containing desired bands would be visualized. (**c**) Representative histology of lung tumors from WT mice and *Cdc42* ^L/L^ mice at 80 weeks post Ad-cre treatment. Scale bar = 500 μm. (**d**) Representative pathology of lung tumors from *Kras* and *Kras/Cdc42* mice at 16 weeks post Ad-Cre treatment. The areas in the boxes of left photos were amplified on the right. Scale bar (left) = 500 μm, Scale bar (right) = 100 μm (**e,f**) Statistical analyses of the number of alveolar and bronchiolar tumors (**e**) and the percentage of bronchiolar tumors (**f**) in *Kras* and *Kras/Cdc42* mice at 16 weeks post Ad-Cre treatment. Al: alveolar; Br: Bronchiolar. Data were shown as mean ± s.e.m. *P < 0.01***P < 0.001. (**g**) Representative immunostaining of KI-67 in alveolar and bronchiolar tumors from *Kras* and *Kras/Cdc42* mice. Scale bar = 50 μm. (**h**) Statistical analyses of proliferative index by KI-67 immunostraining in bronchiolar and alveolar tumor lesions from *Kras* and *Kras/Cdc42* mice. More than 200 high-power fields (HPF) per mouse were counted. Data were shown as mean ± s.e.m. ***P < 0.001.

To investigate the potential role of CDC42 in lung tumorigenesis, we crossed the conditional *Cdc42* ^*L/L*^ allele with *Lox-Stop-Lox Kras* ^*G12D*^ (hereafter named as *Kras*) allele to get *Lox-Stop-Lox Kras* ^*G12D*^; *Cdc42* ^*L/L*^ allele (hereafter named as *Kras/Cdc42*)^24, 25^. Mice were then inoculated with Cre-expressing adenovirus (Ad-Cre) by nasal inhalation as previously described^25^, and analyzed at a series of time points (Fig. 1a). In this model, it remains possible that multiple pulmonary cell lineages are infected with Ad-Cre and neoplastic lesions arise from both bronchiole and alveoli epithelial cells^25^. We initially confirmed the *Cdc42* deletion in lung tumors derived from *Kras/Cdc42* mouse model (Fig. 1b, Supplementary Figs S1–2). As the control, deletion of *Cdc42* alone did not result in any tumor formation over 70 weeks post Ad-Cre treatment (Fig. 1c). Consistent with the essential role of CDC42 in promoting cell division and neoplastic transformation^2, 26^, *Cdc42* loss significantly decreased the lesion number and percentage of alveolar tumors in *Kras/Cdc42* mice (Fig. 1d–f). Surprisingly, we observed a significant increase of the lesion number and percentage of bronchiolar tumors in this model (Fig. 1d–f), featured with the papillae protrusion into airway lumens (Fig. 1d). These bronchiolar lesions in *Kras/Cdc42* model exhibit a high cell proliferating index (presented by KI67 staining) compared with those in *Kras* model (Fig. 1g,h). This analysis demonstrated that *Cdc42* loss increased formation of bronchial and bronchiolar epithelial tumors, but decreased *Kras*-induced tumor formation in alveoli.

### *Cdc42* loss disrupts bronchiole cell polarity

**Figure 2:**
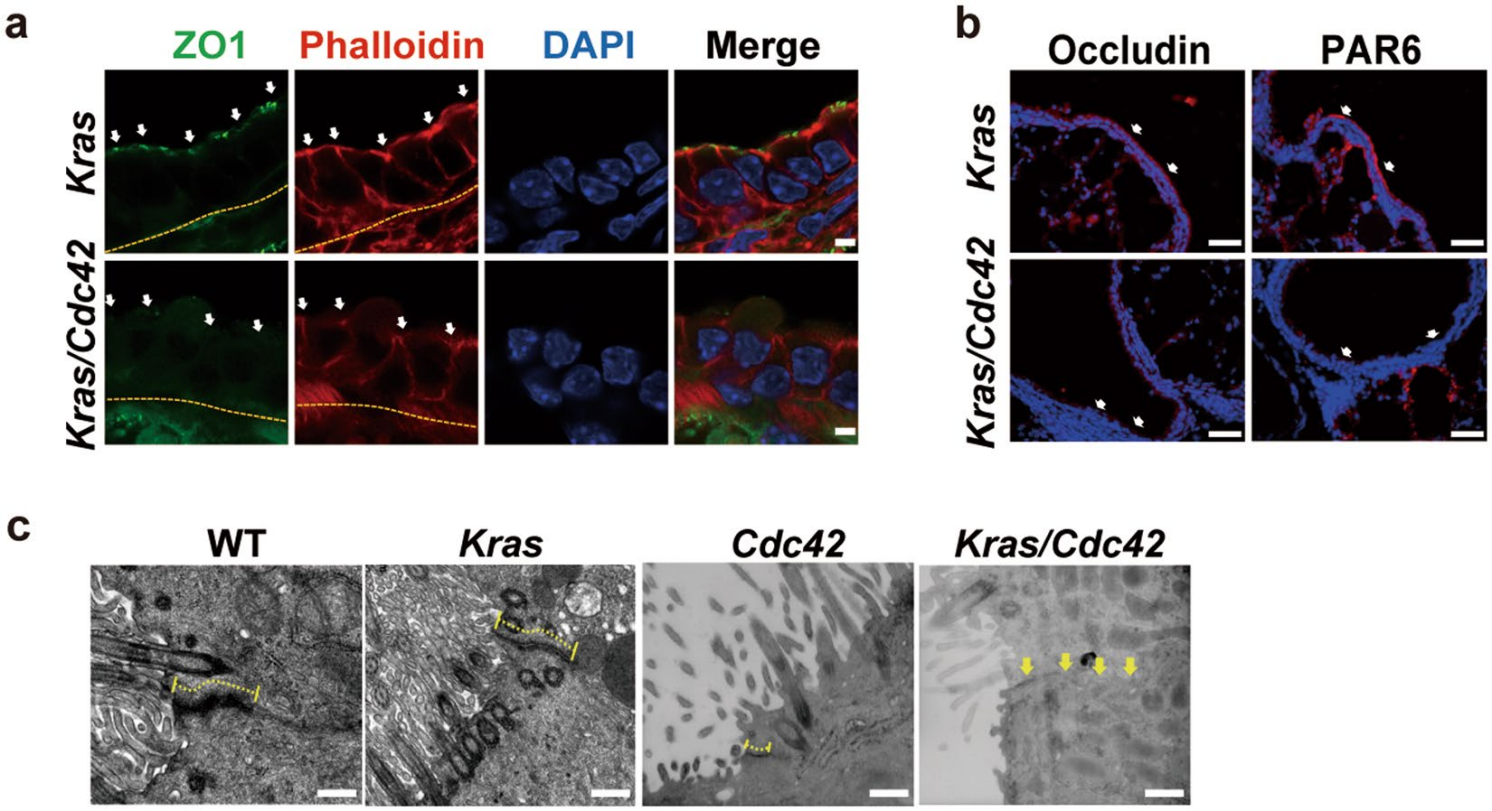
*Cdc42* loss disrupts bronchiole cell polarity. (**a**) Immunofluorescent co-staining of ZO1 (Green), Phalloidin (Red) and 4′,6-diamidino-2-phenylindole (DAPI, blue) in *Kras* and *Kras/Cdc42* mice at 3 weeks post Ad-Cre treatment. The arrows indicate tight junctions at apical surface of bronchiole epithelial cells, and the dashed lines indicate basal surface. Scale bar = 3.5 μm. (**b**) Immunofluorescent staining of Occludin and PAR6 in *Kras* and *Kras/Cdc42* mice at 3 weeks post Ad-cre treatment. The arrows indicate apical surface of bronchiole epithelial cells. Scale bar = 50 μm. (**c**) Ultrastructural analysis of tight junction between bronchiole cells in WT, *Kras*, *Cdc42* (dashed line) and *Kras/Cdc42* (arrows) mice at 3 weeks post Ad-Cre treatment. Scale bar = 500 nm.

We then asked how *Cdc42* loss promoted the bronchiole tumor formation. Normal bronchioles are lined by pseudostratified or single layer epithelia which potentially contribute to contact inhibition and act as the important barrier for neoplastic transformation^27, 28^. Since CDC42 plays a central role in establishing and maintaining epithelial polarity which is frequently disrupted during tumor progression, we first analyzed the subcellular localization of a series of polarity proteins in *Kras* or *Kras/Cdc42* bronchioles at three weeks post Ad-Cre treatment. Our data showed a subcellular dislocation of both ZO1 and Phalloidin, markers for tight junction and F-ACTIN assembling respectively, in *Kras/Cdc42* mice bronchioles at three weeks post Ad-Cre treatment (Fig. 2a). Similar dislocation of other polarity components including PAR6 and Occludin was also detectable in *Kras/Cdc*42 mice (Fig. 2b). Moreover, our data from electron microscopy analyses demonstrated that the ultrastructure of tight junction in bronchioles was disrupted in *Kras/Cdc42* mice but not in *Kras* mice (Fig. 2c).

### *Cdc42* loss promotes the proliferation of KRAS-activated polarized cells potentially through disruption of cell-cell contact inhibition

**Figure 3:**
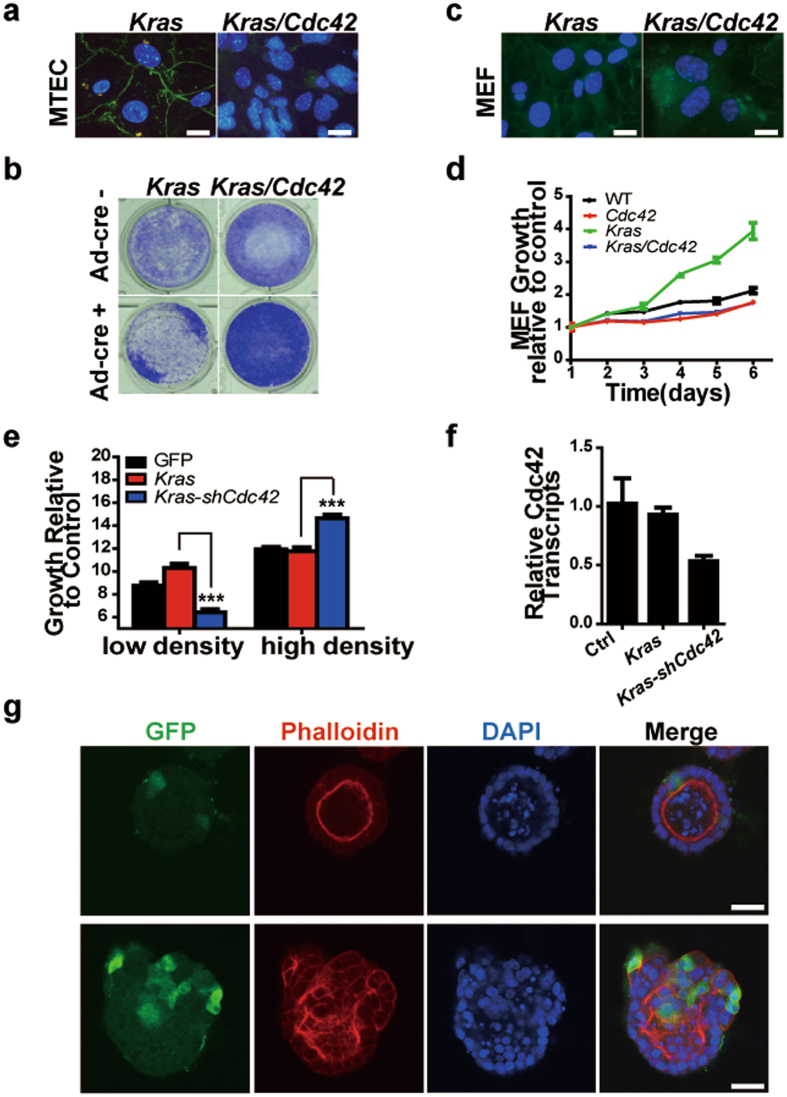
*Cdc42* loss promotes the proliferation of KRAS activated cells potentially through disruption of cell-cell contact inhibition. (**a**) Immunofluorescent staining of ZO1 (Green) and DAPI (blue) in Ad-cre treated MTEC derived from *Kras* and *Kras/Cdc42* mice. Scale bar = 50 μm. (**b**) Crystal violet staining of MTEC derived from *Kras* and *Kras/Cdc42* mice at 72 h with or without Ad-Cre treatment. (**c**) Immunofluorescent staining of ZO1 (Green) and DAPI (blue) in Ad-cre treated MEF derived from *Kras* and *Kras/Cdc42* mice. Scale bar = 50 μm. (**d**) Growth of MEF derived from indicated mice determined by MTT assay during 6 continuous days of cell culture. **(e)** MTT assay indicated growth of MDCK cell line transfected with indicated vector at 72 h (low density) and 120 h (high density) relative to control (12 h). ***P < 0.001. **(f)** Quantitative PCR showing the knockdown efficiency of indicated vector in MCDK. **(g)** Immunofluorescent double staining of Phalloidin (Red) and DAPI (blue) in 3D cultured MDCK expressing *KRAS-copGFP* (top) or *KRAS-shCdc42-copGFP* (bottom). Scale bar = 20 μm.

To test if *Cdc42* loss indeed promotes lung bronchiolar tumorigenesis through disruption of polarity, we utilized two types of cells with distinct polarities including mouse trachea epithelial cells (MTEC) and mouse embryonic fibroblasts (MEF) to analyze the functional consequence of *Cdc42* deletion. In comparison with KRAS activation alone, deletion of CDC42 together with KRAS activation almost completely disrupted the tight junction in MTEC, indicated by ZO1 staining (Fig. 3a), and dramatically increased cell growth (Fig. 3b). On the contrary, in MEF cells which did not form tight junction, *Cdc42* deletion abrogated the promotive effect of KRAS upon cell proliferation (Fig. 3c,d).

We further took advantage of Madin-Darby canine kidney (MDCK) cells, which is widely used for polarity study. Our data showed that *Cdc42* knockdown remarkably inhibited KRAS-activated MDCK cell growth at low cell density but the cell growth was significantly enhanced after reaching 100% confluence (Fig. 3e,f). These data clearly demonstrated that *Cdc42* loss plays opposite roles in cell proliferation, depending on whether cell-cell junction exists or not.

The three-dimensional (3D) system is an excellent model of epithelial morphogenesis in which cells embedded in matrigel form acini, a spherical monolayer enclosing a central lumen. To study the potential role of CDC42 in restricting neoplastic transformation, we infected pre-formed MDCK acini (day 6) with a low dose of lenti-virus expressing *Kras* ^*G12D*^ with or without *Cdc42* knockdown. Consistent with previous study, KRAS activated single cell remained quiescent in growth-arrested acini, with apical location of ZO1 and Phalloidin (Fig. 3g, upper panel). In contrast, single cell with *Cdc42* knockdown in context of KRAS activation initiated inner-growing cell mass from the epithelial layer with disordered ZO1 and Phalloidin localization (Fig. 3g, bottom panel), which morphologically resembled papillae protruding into the airway lumens in *Kras/Cdc42* mice bronchiole (Fig. 1d). These results substantiated the key role of CDC42 and polarity integrity in inhibiting tumor formation.

### Lineage-specific deletion of *Cdc42* in *Kras*-driven lung cancer mouse model

**Figure 4:**
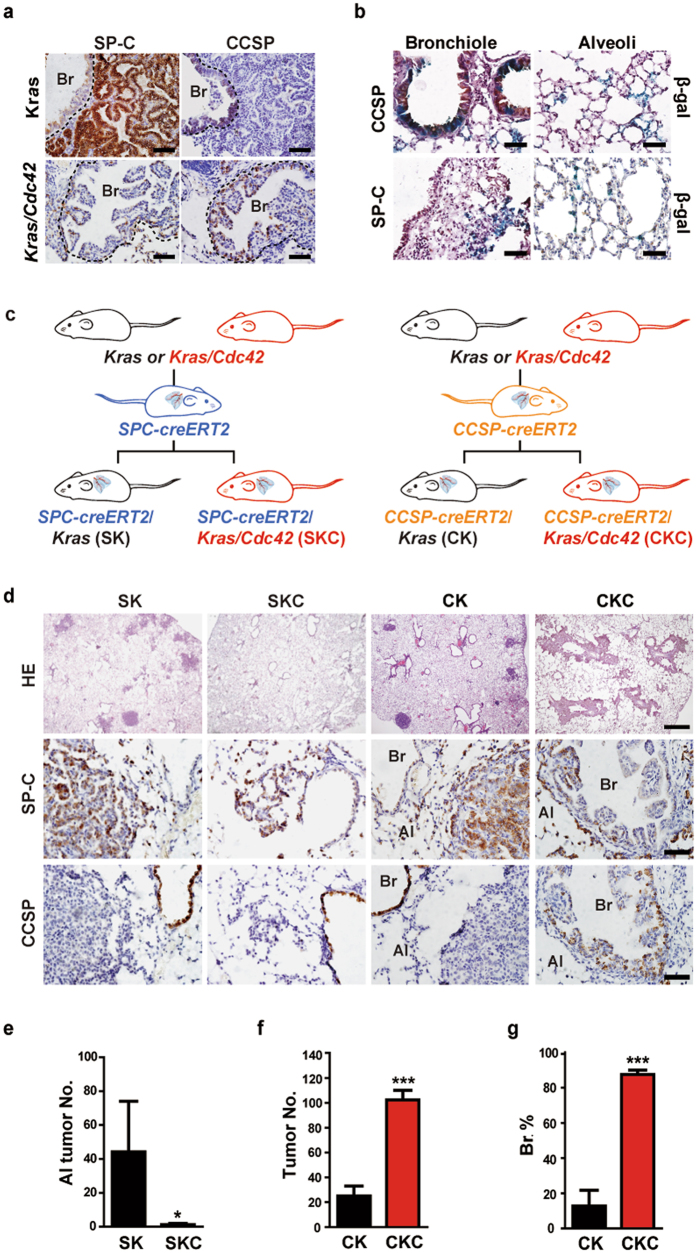
Lineage-specific deletion of *Cdc42* in Club cells and AECII in *Kras* mouse model. (**a**) Immunohistochemical staining for SP-C and CCSP in lung tumors from *Kras* and *Kras/Cdc42* mice. The dashed line indicated the bronchiole. Br: Bronchiole. Scale bar = 50 μm. (**b**) Representative photos of *CCSP-CreERT2/Rosa26R -LacZ* (upper panel) or *SPC-CreERT2/Rosa26R-LacZ* (lower panel) mice lung sections at 4 weeks post tamoxifen administration. β-galactosidase staining (blue) indicates targeted cell. The upper panel is co-stained with CCSP and the bottom is co-stained with SP-C. Scale bar = 50 μm. (**c**) A brief scheme of mouse crossing of *SPC-CreERT2* and *CCSP-CreERT2* allele with *Kras* or *Kras/Cdc42* mice. (**d**) Representative pathology and immunohistochemical staining for SP-C and CCSP of lung tumors derived from *SPC-Cre-ERT2/Kras* (SK), *SPC-Cre-ERT2/Kras/Cdc42* (SKC)*, CCSP-Cre-ERT2/Kras* (CK) and *CCSP-Cre-ERT2/Kras /Cdc42* (CKC) mice at 8 weeks post Ad-Cre treatment. Br: Bronchiole; Al: Alveoli. Scale bar = 500 μm for H&E, Scale bar = 50 μm for immunostaining. (**e**) Statistical analyses of the number of alveolar and bronchiolar tumors found in SK and SKC mice. Data were shown as mean ± s.e.m. *P < 0.05. (**f,g**) Statistical analyses of the number of alveolar and bronchiolar tumors (**f**) and the percentage of bronchiolar tumors (**g**) found in CK and CKC mice. Data were shown as mean ± s.e.m. ***P < 0.001.

Mouse bronchiolar and alveolar tumors are considered to be derived from Club cells and type II alveolar epithelial cells (AECII) respectively^29, 30^. CCSP and the surfactant protein-C (SP-C) are commonly-used markers to distinguish Club cell and AECII^25^. We found that the bronchiolar tumors from *Kras/Cdc42* model were stained positive for CCSP but negative for SP-C, whereas the alveolar tumors were opposite (Fig. 4a). Based on these data, we hypothesize that CDC42 might play a cell type-specific role in lung tumorigenesis.

We then utilized lineage specific ablation of *Cdc42* to dissect the potential roles of *Cdc42* in bronchiolar and alveolar tumor formation. For this, we first constructed the *SPC-Cre-ERT2* (mainly targeting AECII cells) and *CCSP-Cre-ERT2* transgenic alleles (mainly targeting Club cells in bronchiolar epithelia^31^) (Fig. 4b). Given that the *CCSP-Cre-ERT2* strain was novelly constructed, we crossed these two alleles to *Rosa26R-LacZ* reporter mouse strain^32^ and found that tamoxifen-induced LacZ expression from Rosa26 locus was mainly restricted to Club cells that located at bronchioles, and AECII cells located at alveoli, respectively (Fig. 4b). These data are consistant with our previous study^33^. We then crossed *SPC-Cre-ERT2* or *CCSP-CreERT2* allele with either *Kras* or *Kras/Cdc42* mice to generate *SPC-Cre-ERT2/Kras* (SK)^34^, *SPC-Cre-ERT2/Kras/Cdc42* (SKC)*, CCSP-Cre-ERT2/Kras* (CK) and *CCSP-Cre-ERT2/Kras/Cdc42* (CKC) mice and performed the detailed tumor analyses after tamoxifen treatment (Fig. 4c). As expected, the majority of tumors in the SK model were located at alveoli; SKC mice demonstrated significantly decreased tumorigenesis (alveolar tumors) compared to the SK group (Fig. 4d,e). On the other hand, we found that the majority of lung tumors in CKC mice were located at bronchioles, and stained positive for CCSP but negative for SP-C (Fig. 4d). Interestingly, a dramatic increase of tumor formation mainly in bronchiole was detected in CKC mice compared to CK mice (Fig. 4d,f,g). Less than 20% bronchioles tumor was found in CK mice, suggesting that KRAS^G12D^ activation alone is difficult to transform the bronchiole epithelium cells with intact cell polarity.

Collectively, these data support that CDC42 functions oppositely in different lineage tumor formation: it promotes tumor growth in alveoli while prevents tumor formation in bronchioles.

### High CDC42 level correlates with SP-A level in human lung adenocarcinoma

**Figure 5:**
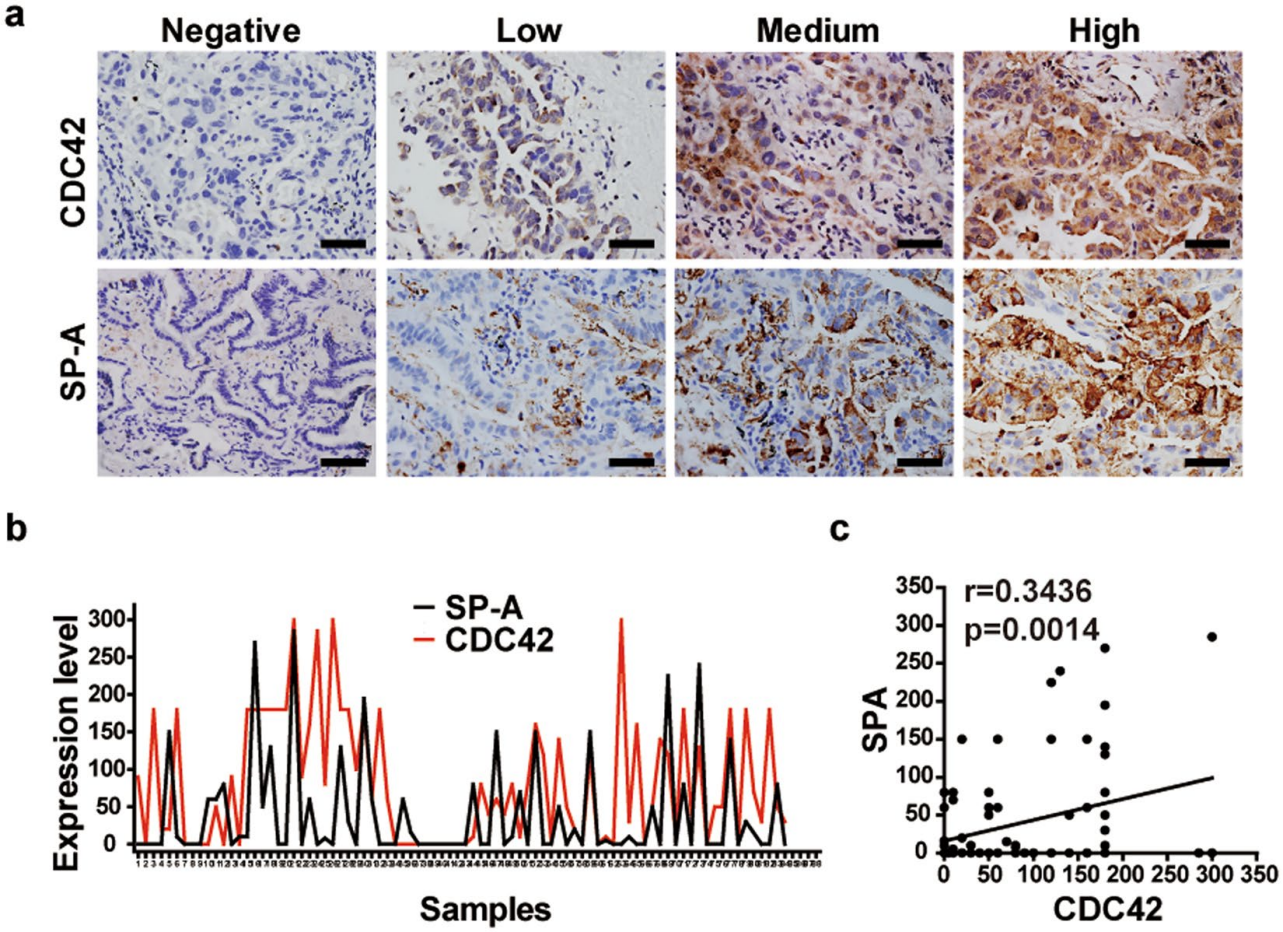
CDC42 expression positively correlates with alveolar marker SP-A expression in human lung adenocarcinoma. (**a**) Representative group (low, medium, high) of immunohistochemical staining for SP-C and CDC42 in 84 human lung adenocarcinoma samples. Scale bar = 50 μm. (**b**) Expression level of CDC42 or SP-A in 84 human lung adenocarcinoma samples. Scale bar = 50 μm. (**c**) Pearson correlation analysis showed a significant positive correlation between CDC42 expression and SP-A expression (P < 0.01, r = 0.3436) in 84 human lung adenocarcinoma. Full line represents linear regression of data.

To further study the potential clinical relevance of our findings from mouse model, we analyzed a set of 84 lung adenocarcinoma for expression patterns of CDC42 and SP-A, a marker for human AECII^35^. Immunohistochemistry staining were performed and blindly scored according to Allred score system (Fig. 5a). Consistent with previous study^9^, we found that 57.5% of tumors were positive for CDC42 expression. Pearson correlation analysis showed that CDC42 level positively correlated with SP-A level (Fig. 5b,c; r = 0.3436, P = 0.0014). Although SP-A is a biomarker for normal human AECII, human lung cancer frequently lost the expression of lineage biomarkers. Thus, our clinical data analyses only provide an implication that CDC42 expression might be specifically high in those lung cancer derived from AECII.

## Discussion

In this study, we have identified an unexpected cell type-dependent role of CDC42 in lung tumorigenesis. In AECII, *Cdc42* loss strongly prevents Kras-driven neoplastic transformation, establishing the tumor-promotive role of CDC42. These results are consistent with early studies carried out in various mammalian cell lines by overexpressing dominant-negative and/or constitutively active mutants showing the necessity of CDC42 in transformation^11, 12, 36^. In contrast, our data shows that *Cdc42* loss not only disrupted epithelial cell polarity and branching morphogenesis of the developing lung as previously described^37^, but also promotes KRAS-induced overgrowth and tumor formation in adult Club cells.

**Figure 6:**
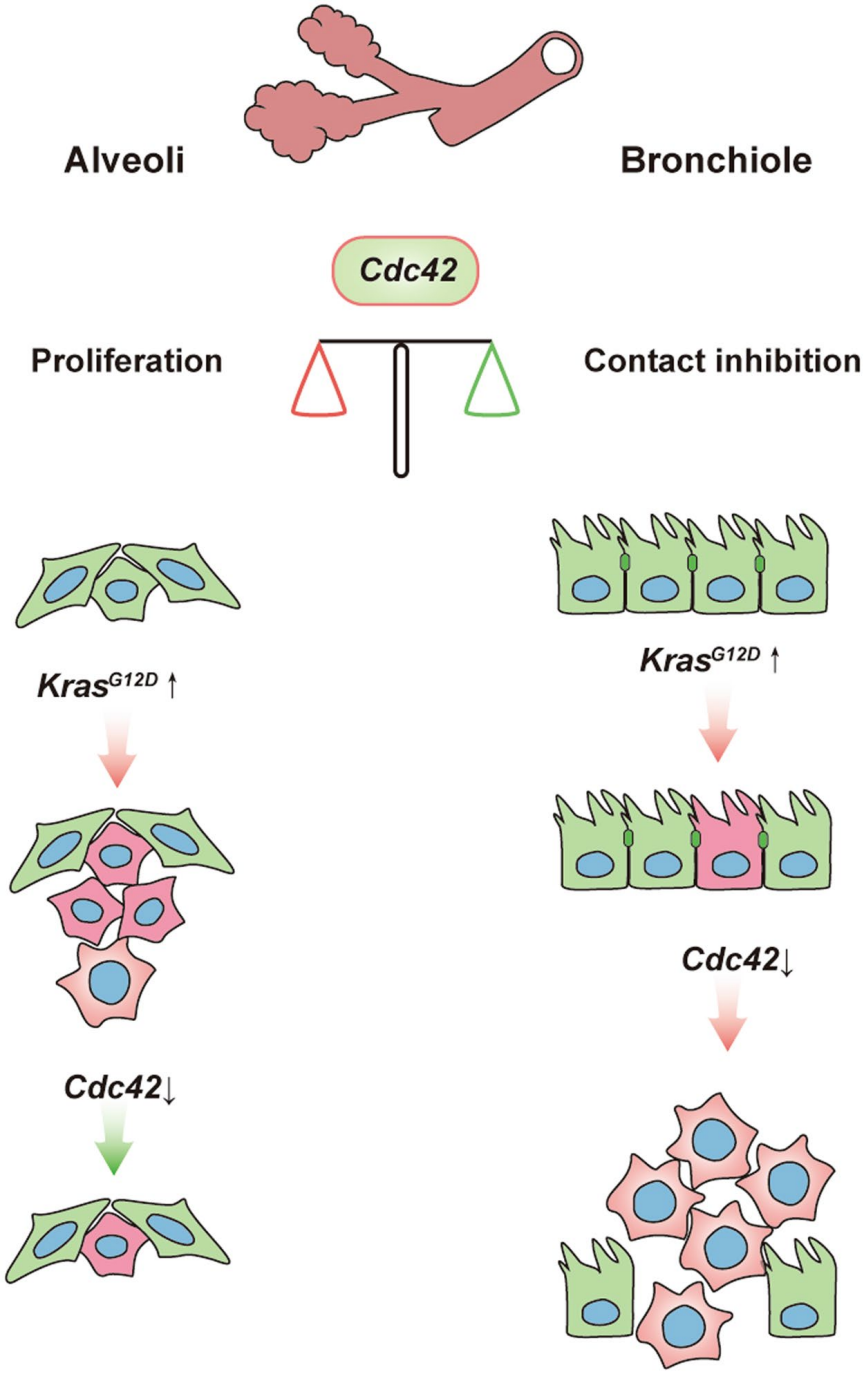
Schematic illustration of the function of CDC42 in lung cancer. In this study, we have identified an unexpected cell type-dependent role of CDC42 in lung tumorigenesis. In type II alveolar epithelial cells (AECII), *Cdc42* loss strongly prevents *Kras*-driven neoplastic transformation, establishing the tumor-promotive role of CDC42. The pseudostratified or single-layer polarized Club cells, are separated by apical junctions which function as physical barrier by providing a tight seal between the membranes of the neighboring cells. These internal physical barriers of the neighboring cells function as a non-cell-autonomous tumor suppressor to restrict Kras-induced cell proliferation and prevent bronchiole tumor formation. Loss of core polarity protein CDC42 disrupted cell polarity and cell-cell contact inhibition, thereby leading to Kras induced bronchiole papillary hyperplasia. Green: normal cells; Pink: genetically mutated cells.

Our results further suggest that *Cdc42* loss induces *Kras*-derived bronchiole tumor formation potentially through disruption of cell polarity and cell-cell contact inhibition. Compared with alveolar cells, bronchiolar epithelia cells are close-packed and express polarity-related protein specifically located on apical or basal, which function as a non-cell-autonomous tumor suppressor to restrict KRAS-induced cell proliferation. Since CDC42 plays an essential role in regulation of cell polarity in these cells, its deletion leads to disrupted cell-cell junction, the loss of contact inhibition, eventually triggers cell overgrowth. Together, these data suggest that the cell polarity maintained by CDC42 might serve as an important barrier for bronchiolar tumor formation in lungs. Our findings are consistent with Previous studies showing that the dysfunction of certain polarity genes is associated with tumor initiation or progression^38–40^.The role of CDC42 in cell proliferation (mainly in alveoli) and contact inhibition (mainly in bronchiole) during the tumorigenesis is summarized in Fig. 6.

In conclusion, our data show that CDC42 functions in a cell-type specific manner in lung tumorigenesis in context with different cell lineages. Loss of *Cdc42* promoted KRAS-induced Club cell tumor formation, underscoring an important tumor initiation function of polarity loss which was previously considered a by-product of abnormal cell accumulation. In addition, studies from mouse model and cell lines can be further applied to human pathological conditions. Of course, more detailed studies are needed to determine the feasibility and therapeutic benefit of targeting CDC42 in human disease.

## Materials and Methods

### Mouse treatment

*Kras* ^*G12D*^, and *Rosa26-LacZ* mice were originally generously provided by Dr. T. Jacks and Dr. L. Cheng, respectively. *Cdc42* ^*L/L*^ mice were generated as described before^24, 41^. The *SPC-CreERT2* allele was made as previously reported^34^. The *CCSP-CreERT2* allele was made using the rat *CCSP* promoter^31^ driving the *CreERT2* gene expression with similar strategy as previously reported for *SPC-CreERT2* allele^34^. Progeny were screened using southern blot and confirmed by PCR strategy. All mice were housed in a specific pathogen-free environment at the Shanghai Institute of Biochemistry and Cell Biology and treated in strict accordance with protocols approved by the Institutional Animal Care and Use Committee of the Shanghai Institutes for Biological Sciences, Chinese Academy of Sciences. All experiments were performed in accordance with relevant guidelines and regulations. For *Kras*, *Kras/Cdc42* lung cancer mice model, mice were treated with 2 × 10^6^ plague-forming units (PFU) of Ad-Cre (purchased from University of Iowa adenoviral core) or lenti-virus through nasal inhalation at 6~8 weeks of age^25^. For *SPC-Cre-ERT2/Kras* (SK), *SPC-Cre-ERT2/Kras/Cdc42* (SKC)*, CCSP-CreERT2/Kras* (CK) or *CCSP-CreERT2/Kras/Cdc42* (CKC) mouse models, tamoxifen in sunflower oil (40 mg/kg) were given to mice via intraperitoneal injection daily for five continuous doses. Mice were sacrificed at a serial time points for gross inspection, histopathological examination and molecular analyses. The numbers of lung bronchiolar and alveolar tumors were analyzed according to pathology for different mouse cohorts and the percentage of regional tumors was then calculated accordingly.

### Histopathological Analysis

Histopathological analysis was performed as described before^25^. Briefly, mice were sacrificed and lung tissues were inflated and fixed in PFA, embedded in paraffin or frozen in optimal cutting temperature compound (OCT) and sectioned for hematoxylin and eosin (H&E) staining. Immunohistochemical and immunofluorescence analyses were performed as described^34^. Antibodies against the following proteins were used: KI-67 (Novocastra Laboratories Ltd), CDC42 (Abcam), ZO1 (33-9100, Zymed); PAR6 (sc-14405, Santa Cruz), Occludin (353197a Zymed), Phalloidin (R415 Invitrogen), CCSP (Santa Cruz), SP-C (AB3786, Chemicon), Beta Galactosidase (ab9631, Abcam), Biotinylated goat anti-rabbit secondary antibody (ZYMED company), Alexa Fluor 555 or 488 conjugated anti-mouse, rat or rabbit IgG secondary antibodies (Invitrogen).

### β-galactosidase activity analyses

For β-galactosidase activity assays (X-gal staining), mouse lungs were isolated and immediately incubated for 2 hours in a 20-fold volume of ice-cold fixative (1% formaldehyde, 0.2% gluteraldehyde and 0.02% NP40 in PBS) at 4 °C on a rolling platform. The fixative was removed and the tissues were washed twice in PBS for 20 min at room temperature. The galactosidase substrate (5 mM K_3_Fe (CN)_6_, 5 mM K_4_Fe (CN)_6_, 2 mM MgCl_2_, 0.02% NP40, 0.1% sodium deoxycholate and 1 mg/ml X-gal in PBS) was then added and the tissues were incubated in the dark overnight. The stained tissues were transferred to tissue cassettes and paraffin blocks were prepared following standard methods.

### Constructs, Cell culture and *in vitro* Growth Characteristics

The ORF of *Kras* gene was amplified from human cDNAs, and point mutated at G12D following protocol of STRATAGENE QuikChange ® XL Site-Directed Mutagenesis Kit. The *Kras* ^*G12D*^ and the shRNAs towards human CDC42 was ligated into expression vectors *pCDH-EF1-copGFP* (Systems Biosciences). The target sequence is: shCdc42: 5′-GCCTATCACTCCAGAGACT-3′.

### Mouse embryonic fibroblasts (MEF)

Embryonic day 13.5 embryos were dissected from *Kras/Cdc42* intercrossed females, and MEF were isolated and maintained in DMEM (Hyclone) medium supplemented with 10% fetal bovine serum (FBS, Biochrom, AG), penicillin and streptomycin. MEF (~4 × 10^5^) at 6-well plate were virally infected with Ad-Cre (4 × 10^6^ CFU) overnight and then changed with fresh medium. MDCK and HEK293T cells (ATCC) were cultured in DMEM with 10% FBS. Viral infection of cells was as previously described^42^.

For *in vitro* growth assay, cells were seeded in 96-well plates (2500 cells per well), and the proliferation information were obtained by MTT (3–4, 4-dimethylthiazol-2, 5 diphenyl tetrabromide) assay every day as previously described^43^.

### Mouse tracheal epithelial cell culture (MTEC)

MTEC harvest was performed as previously described^44^. Briefly, 8–10 wk *Kras* or *Kras/Cdc42* mice were sacrificed and immersed in 70% ethanol (avoiding airway submersion), and tracheas were resected from the larynx to the bronchial main branches and collected in ice-cold Ham’s F-12 pen-strep. After overnight digestion of 0.25% trypsin at 4 °C, the suspended trachea cells were collected for incubation in tissue culture plates for 3–4 hrs in 5% CO_2_ at 37 °C to adhere fibroblasts, and nonadherent cells were collected by centrifugation, resuspended in MTEC/Plus medium, and maintained in collagen coated 24-well plate. After 72 hrs growth, MTEC (~1 × 10^5^) at 24-well plate were virally infected with Ad-Cre (1 × 10^6^ CFU) overnight and then changed with fresh medium. Immuno or crystal violet staining was performed after another 4 days’ growth.

### Electron microscopy

After the mice were sacrificed, small fragments (~1 mm^3^) of lung were fixed overnight in a solution containing 4% paraformaldehyde, 2.5% gluteraldehyde in 0.1 M PBS (pH 7.4). Tissue fragments were washed for 20 min in 0.1 M PBS for three times and subsequently treated with 2% osmium tetroxide in PBS for 1.5 hrs. After washing for 5 min × 3 with PBS, specimens were dehydrated in a graded ethanol series and embedded in Epon. For orientation purposes, 1 µm sections were stained with toluidine blue. Ultrathin sections (70–80 nm) were then cut with a Reichert–Jung ultramicrotome, collected on formvar coated nickel grids, stained with uranyl acetate for 10 min and with lead citrate for 7 min. Samples were examined with a FEI Tecnai G2 Spirit Transmission Electron Microscope.

### Human Lung Cancer Specimen analyses

A total of 84 NSCLC patient samples were collected with the approval by the institutional review committees of Shanghai Cancer Center, Fudan University. Patients gave written informed consents. The specimens were used for immunostaining of SP-A and CDC42 and analyzed for clinical relevance. All human methods were performed in accordance with the relevant guidelines and regulations.

### Statistical Analysis

Data were analyzed by Student’s *t* test or Pearson correlation test; *P* < 0.05 was considered significant.

### Data availability

All data generated or analysed during this study are included in this published article.

## Electronic supplementary material

Supplementary information
