# Supplementary material for: Cell Division Cycle 42 plays a Cell type-Specific role in Lung Tumorigenesis
Source: Sci Rep. 2017 Sep 4;7:10407. doi: 10.1038/s41598-017-10891-0 (PMC5583260; doi:10.1038/s41598-017-10891-0)
Supplement: Supplementary file 1 — Supplementary information [file 41598_2017_10891_MOESM1_ESM.pdf]

Supplementary information

Cell Division Cycle 42 plays a Cell type-Specific role in Lung Tumorigenesis

Chao Zheng, Yuetong Wang, Liu Yang, Shuhua Zhou, Yijun Gao, Fuming Li, Yan Feng, Zuoyun Wang, Lixing Zhan, Qin Yan, Xueliang Zhu, Kwok-Kin Wong, Zhengjun Chen, Hongbin Ji

Content

Figure S1. PCR confirm *Cdc42* deletion in lung tumors derived from *Kras/Cdc42* mouse.

Figure S2. Western blot confirm *Cdc42* deletion in lung tumors derived from *Kras/Cdc42* mouse.

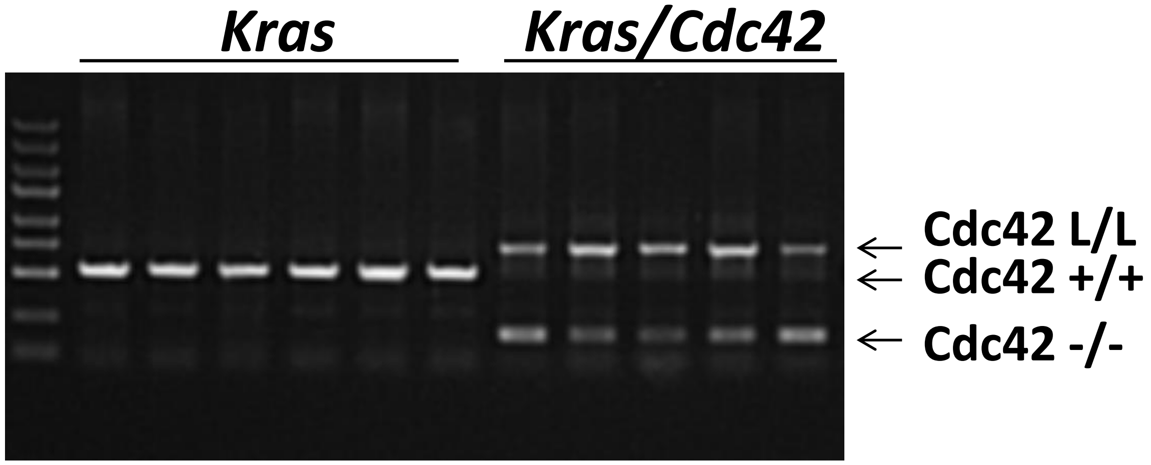

Figure S1. PCR confirm *Cdc42* deletion in lung tumors derived from *Kras/Cdc42* mouse. Full-length PCR gel of low contrast is shown with conditional *Cdc42* allele recombination in tumors from *Kras* and *Kras/Cdc42* mice

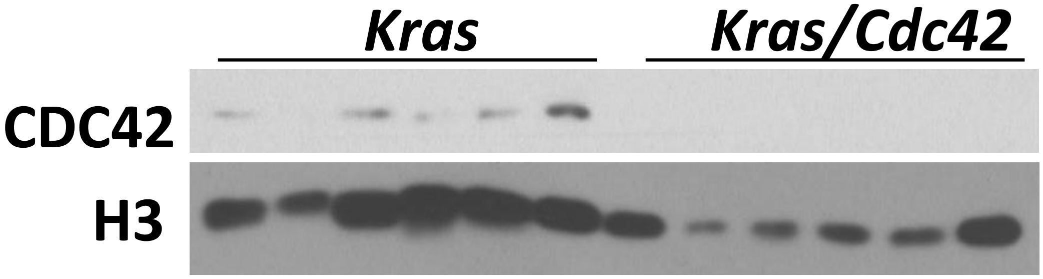

Figure S2. Western blot confirm *Cdc42* deletion in lung tumors derived from *Kras/Cdc42* mouse. Western blot of CDC42 expression in tumors from *Kras* and *Kras/Cdc42* mice (low-exposure). Histone 3 (H3) serves as a loading control. The cropped blots are used in the figure. The membranes were cut prior to exposure so that only the portion of gel containing desired bands would be visualized.
